# Supplementary material for: Original Basic Activation for Enhancing Silica Particle Reactivity: Characterization by Liquid Phase Silanization and Silica-Rubber Nanocomposite Properties
Source: Polymers (Basel). 2022 Apr 20;14(9):1676. doi: 10.3390/polym14091676 (PMC9105500; doi:10.3390/polym14091676)
Supplement: Supplementary file 1 [file polymers-14-01676-s001.zip › polymers-1668815-supplementary.pdf]

Original basic activation for enhancing silica particles reactivity: characterization by liquid phase silanization and silica-rubber nanocomposite properties

Enzo Moretto[1], Chuanyu Yan[1], Reiner Dieden[1], Pascal Steiner[2], Benoît Duez[2], Damien Lenoble[1], Jean-Sébastien Thomann[1,] *

[1] MRT Department, Luxembourg Institute of Science and Technology, 41 Rue du Brill, Belvaux, L-4422 Luxembourg, Luxembourg; enzo.moretto@list.lu (E.M.), chuanyu.yan@list.lu (C.Y.), reiner.dieden@list.lu (R.D.), damien.lenoble@list.lu (D.L.)
[2] Goodyear S.A, Avenue Gordon Smith, Colmar-Berg, L-7750 Luxembourg, Luxembourg; benoit_duez@goodyear.com (B.D.); pascal_steiner@goodyear.com (P.S.)
* Correspondence: jean-sebastien.thomann@list.lu (J.-S.T.)

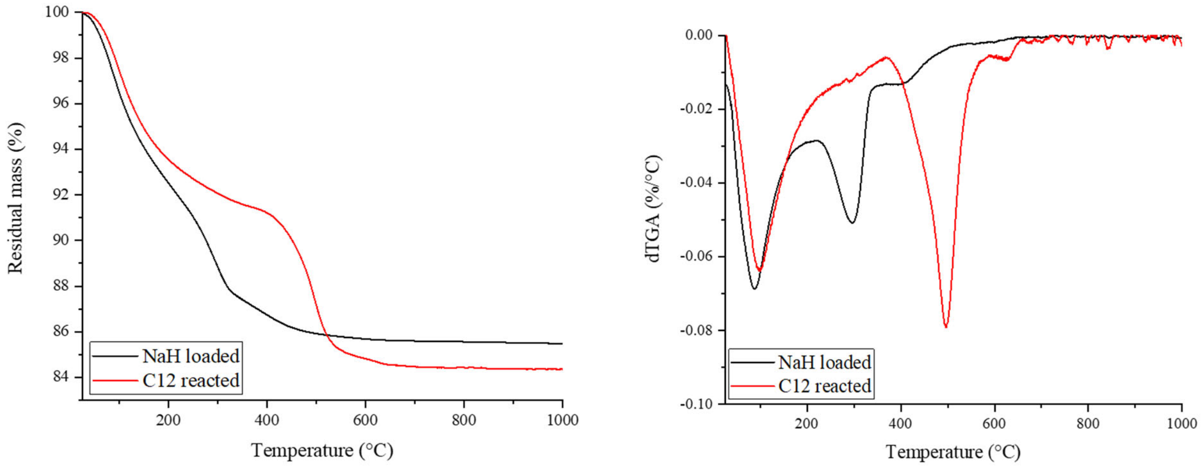

Figure S1 – Thermogram (left) and dTGA (right) curves of NaH activated silica (black) and NaH-C12 reacted silica (red)

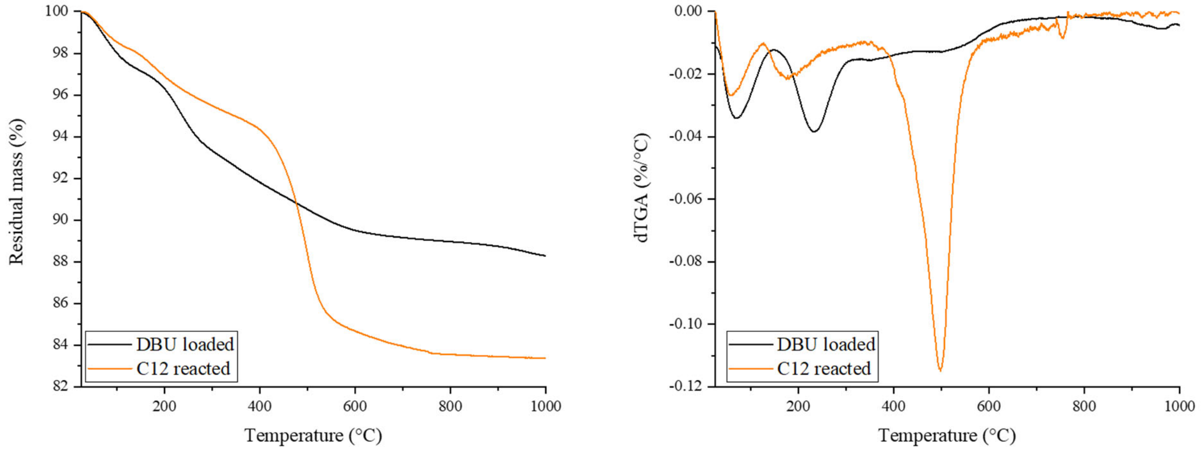

Figure S2 – Thermogram (left) and dTGA (right) curves of DBU-activated silica (black) and DBU-C12 reacted silica (orange)

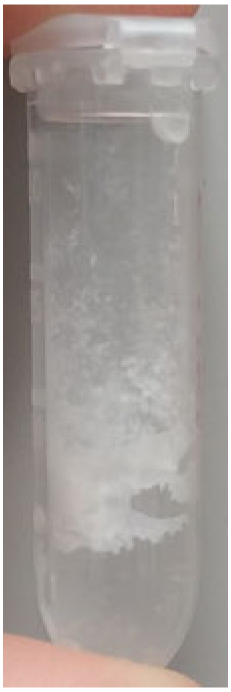

Figure S3 – Photograph of the white residual film after reaction of C12 silane with $CsF/DCl/D_2O$ solution
